# Systematically Differentiating Functions for Alternatively Spliced Isoforms through Integrating RNA-seq Data

**DOI:** 10.1371/journal.pcbi.1003314

**Published:** 2013-11-07

**Authors:** Ridvan Eksi, Hong-Dong Li, Rajasree Menon, Yuchen Wen, Gilbert S. Omenn, Matthias Kretzler, Yuanfang Guan

**Affiliations:** 1 Department of Computational Medicine and Bioinformatics, University of Michigan, Ann Arbor, Michigan, United States of America; 2 Department of Internal Medicine, University of Michigan, Ann Arbor, Michigan, United States of America; 3 Department of Electrical Engineering and Computer Science, University of Michigan, Ann Arbor, Michigan, United States of America; University of California San Diego, United States of America

## Abstract

Integrating large-scale functional genomic data has significantly accelerated our understanding of gene functions. However, no algorithm has been developed to differentiate functions for isoforms of the same gene using high-throughput genomic data. This is because standard supervised learning requires ‘ground-truth’ functional annotations, which are lacking at the isoform level. To address this challenge, we developed a generic framework that interrogates public RNA-seq data at the transcript level to differentiate functions for alternatively spliced isoforms. For a specific function, our algorithm identifies the ‘responsible’ isoform(s) of a gene and generates classifying models at the isoform level instead of at the gene level. Through cross-validation, we demonstrated that our algorithm is effective in assigning functions to genes, especially the ones with multiple isoforms, and robust to gene expression levels and removal of homologous gene pairs. We identified genes in the mouse whose isoforms are predicted to have disparate functionalities and experimentally validated the ‘responsible’ isoforms using data from mammary tissue. With protein structure modeling and experimental evidence, we further validated the predicted isoform functional differences for the genes *Cdkn2a* and *Anxa6*. Our generic framework is the first to predict and differentiate functions for alternatively spliced isoforms, instead of genes, using genomic data. It is extendable to any base machine learner and other species with alternatively spliced isoforms, and shifts the current gene-centered function prediction to isoform-level predictions.

## Author Summary

In mammalian genomes, a single gene can be alternatively spliced into multiple isoforms which greatly increase the functional diversity of the genome. In the human, more than 95% of multi-exon genes undergo alternative splicing. It is hard to computationally differentiate the functions for the splice isoforms of the same gene, because they are almost always annotated with the same functions and share similar sequences. In this paper, we developed a generic framework to identify the ‘responsible’ isoform(s) for each function that the gene carries out, and therefore predict functional assignment on the isoform level instead of on the gene level. Within this generic framework, we implemented and evaluated several related algorithms for isoform function prediction. We tested these algorithms through both computational evaluation and experimental validation of the predicted ‘responsible’ isoform(s) and the predicted disparate functions of the isoforms of *Cdkn2a* and of *Anxa6*. Our algorithm represents the first effort to predict and differentiate isoforms through large-scale genomic data integration.

## Introduction

Determining the functions of proteins is a central goal of genetics, fundamental for understanding the molecular basis of diverse genetic diseases [1]–[7]. During the past few decades, significant efforts have been made to integrate and develop diverse machine learning algorithms for gene function prediction through large-scale genomic data integration [8]–[12], such as Support Vector Machines, Bayesian classifications and Artificial Neural Networks. Despite differences in implementation and performance of the specific algorithms, the essence of these methods for gene function prediction is ‘supervised learning’, in which a model of features derived from functional genomic data (such as microarray, protein-protein physical interactions, gene-gene genetic interactions) is constructed to delineate a defined set of ‘positives’ (genes annotated to the function under consideration) and ‘negatives’ (genes without the function). These algorithms have significantly accelerated our understanding of gene functions.

A major intellectual limitation of the current function prediction paradigm is that it considers a gene as a single entity without differentiating the functional diversity of alternatively spliced isoforms. Alternative splicing is a major source of protein molecular function diversity and regulatory diversity. In humans, 95% of multi-exon genes undergo alternative splicing, generating proteins of potential different functions [13]–[21]. For example, TRPM3, which encodes a type of cation-selective channels in human, can be alternatively spliced into two variants targeting different ions [22]–[24]. Other splice variants have been reported with distinctly opposite functions. For example, the splice variants of *BCLX* are antiapoptotic and pro-apoptotic, respectively [25]. Similarly, *CASP3*-L variant is pro-apoptotic while *CASP3*-S is antiapoptotic [26]. Differences in function are sometimes reflected on the regulatory level: two alternatively spliced transcripts of *OSR2* have opposite transcriptional activities, activation and repression [26]. Attempts to capture such differential functions are currently limited to low-throughput experimental approaches and protein domain analysis. However, based on the protein domain annotation we downloaded in Dec, 2012, only 34% of the isoform pairs (of the same gene) in the NCBI database have different domains. The majority of the isoforms of the same gene differ in subtle ways which are not reflected by protein domains. We expect that computational methods developed to differentiate isoform functions through integrating functional genomic data will assist a deeper, high-resolution understanding of gene functions.

The key challenge facing isoform prediction is the lack of a systematic catalog of isoform-level function annotations and large-scale genomic data resolved at the isoform level. The latter is resolved by the unprecedented amount of transcriptomic data generated by next-generation sequencing [27]–[29]. RNA-seq data available in public databases [30] now surpass the number of microarray data that have been previously used to infer functions at gene levels [31]. Algorithms have been developed to assign isoform-level expression values [28], [32]–[39]. They provide a resource for isoform-level features that can be used as input data to infer isoform functions.

However, the fundamental challenge remains: a genome-wide set of ‘ground-truth’ annotations of functions at the isoform level is still lacking. Isoform functions have been computationally inferred through domains, binding regions and individual binding sites [40]–[46]. In widely used databases such as Gene Ontology [47], [48] and KEGG [49], biological functions are defined at the gene level. Under these circumstances, to predict whether a gene is related to a specific function, supervised learning algorithms will be deployed to derive a model from the genomic data we collect. For example, to predict ‘mitochondria biogenesis’-related genes, we need a ‘gold standard’ set of genes known to be related to mitochondria biogenesis, and find out how these genes differ from other genes in their expression patterns. Without an existing and comprehensive set of annotated isoforms, standard ‘supervised learning’ approaches are not applicable to predicting different functions for splice isoforms.

We developed a generic framework that improves gene function prediction and provides information for isoform-level functions. Our framework intends to locate the ‘responsible’ set of isoform(s) of annotated genes of a specific function, through iteratively correcting the composition of this set to maximize its ‘discriminativity’ against the negatives. For example, for mitochondria biogenesis, we have a set of genes *G^+^_1_*, *G^+^_2_*, …, *G^+^_n_* annotated to this function (positive genes). Each gene is considered as a bag of alternatively spliced isoforms. We then try to find out which isoform(s) of this positive set of genes can be selected to maximize the difference between them and the negative isoforms, *i.e.*, the *G^i^_k_^+^*, where *k* is the gene index, and *i* is the isoform index. The isoforms in the *G^i^_k_^+^* set are iteratively updated to maximize the similarity within them. The model derived from the *G^i^_k_^+^* set (instead of *G_k_^+^*) is then used to classify whether an isoform has the biological function, mitochondria biogenesis, in this example. From this perspective, our problem is a multiple instance learning task [50]–[53], in which each example considered positive with respect to certain property contains multiple discrete elements, of which at least one of them must be positive.

We used an iterative algorithm to approximate the solution to the above optimization problem. We found that our algorithm can successfully capture the differential isoform functions as evidenced by prediction accuracy on multi-isoform genes as well as literature and experimental validation on isoforms that are drastically different in their assigned functions. Computationally predicting isoform functions or differentiating functions for isoforms of the same genes in a genome-wide manner using high-throughput genomic data has not been done prior to our work. This framework is also generic. It can be integrated into any basic machine learning algorithm such as logistic regression, random forests or deep learning, and can be readily extended to predict isoform functions in other organisms as well as other properties of isoforms such as phenotypes and interaction networks. Our prediction results are available to the user community online at http://guanlab.ccmb.med.umich.edu/isoPred/.

## Results

We first established the workflow that generates isoform-level function predictions. Then we validated our models with computational cross validation, focusing on performance comparison between multiple-isoform genes and single-isoform genes. The predicted ‘functional’ isoform(s) are further validated using breast cell proteomic data. Finally, through protein structure modeling and experimental evidence, we validated our predictions for *Cdkn2a* and for *Anxa6*, whose isoforms were predicted to be responsible for different functions.

### The isoform function prediction framework

We aim at predicting isoform functions while functional annotations have been based on genes. The model therefore needs to be learned at the isoform level rather than at the gene level (see **Figure S1** for a comparison between a traditional gene function learning problem and our isoform function prediction problem). Our core idea is to model the common patterns of a **subset** of isoforms across genes associated with a particular function, with the requirement that at least one of the isoforms of each positive gene must retain the common feature pattern. This is a multiple instance problem, the aim of which is to identify the hidden labels of the isoforms of the positively annotated genes and use these hidden labels to construct classification models to label additional isoforms.

We used maximum margin-based classification as base-learner in this iterative process [54], due to the success of SVM in the protein function prediction domain [9]. Such a framework could be integrated into any other machine learner, such as deep learning or Bayesian classification.

**Figure 1:**
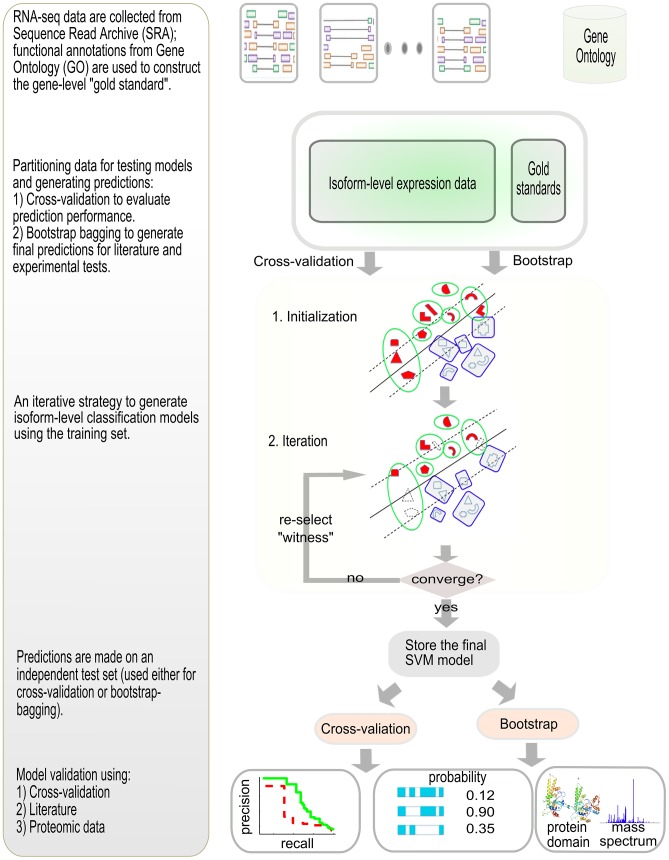
Overview of the computational approach for predicting functions for alternatively spliced isoforms. We collected RNA-seq data from the sequence read archive (SRA) database and estimated isoform-level expression values using state-of-the-art software [32], [34]. We then generated a gene-level gold standard using Gene Ontology (GO) annotations. For each biological function, this gold standard contains positive genes (annotated to the function under investigation) and negative genes (other genes). Our study contains two major parts: cross-validation for performance estimation and bootstrap bagging for generating final predictions as well as performance evaluation. For cross-validation, we partitioned the examples into a training set for model development and a test set for model validation. For generating final predictions for all isoforms, we sampled with replacement to construct a training set, and then used this training set to construct models to assign prediction probabilities to the out-of-bag set. The final predictions for all isoforms were made by calculating the median prediction values of all out-of-bag sets. For each training set, a model was derived from the RNA-seq data to delineate the positives and the negatives. This model was used to classify the training set and update the labels of the isoforms of the positive genes, under the criterion that at least one isoform of a positive gene must remain positive. This new assignment is then used to construct the model in the next iteration. This process is iterated until the assignment of positive isoforms no longer changes, and then the final model was used to assign a prediction value to the test or the out-of-the-bag set. Bootstrap was done for 30 iterations and the median value for each out-of-the-bag isoform was taken as the final prediction value. The predictive performance of our model was assessed through three approaches: (1) cross-validation of gene-level predictive performances, focusing on comparison between single-isoform genes and multiple-isoform genes, (2) literature validation and (3) experimental validation of top predictions using proteomic data.

To elucidate our algorithm, we will use the following definitions throughout the paper:

- A ‘**bag**’ refers to a gene, which consists of multiple isoforms.
- **‘Instances’** refer to individual isoforms.
- A **‘positive’** bag refers to a gene related to the specific function under study.
- **‘Witness(es)’** refer to the isoform(s) of a positive gene related to the specific function under study. A ‘positive’ bag has at least one witness;

Our algorithm aims to identify a subset of isoforms of the positive genes that maximizes the difference between them and the negative isoforms. Identifying the best combination of isoforms from the positive genes is difficult. The ideal solution requires excessive computational time. We therefore approximated the solution with an iterative algorithm (**Figure 1**). For the training set, in the first iteration, we assign every isoform of a positive gene to be positive. We then establish a classifier, with which we can go back to classify the training set. This classifier will assign some isoforms of the positive gene to be positive (the ‘witnesses’), and some to be negative, but at least one isoform of a positive gene must be positive. This subset of ‘witnesses’ is iteratively updated to maximize the inter-class distance. Using this assignment, a new classifier is established, which could again be used to classify the training set. We iterate this process to update the ‘witnesses’. This procedure is repeated until convergence is achieved (**Figure 1**).

Because of the properties and relationships between genes and isoforms, this Multiple Instance Learning (MIL) framework is suitable for our problem. MIL assumes that there are one or more positive instances in a positive bag, and, if we can identify these positive instances in positive bags, we can expect to have a better classifier by excluding remaining instances from the positive class. Biologically, if a gene is annotated to a function, one or more of its isoforms will be selected as witness(es) as the ones that are related to this function, and other isoforms will be marked as non-functional. By excluding these non-functional isoforms, the classifier is expected to be more accurate.

### Implementation and testing parameters

To generate alternatively spliced transcript-level features, we collected transcriptomic data from public RNA-seq experiments (Dataset S1). These RNA-seq datasets cover a wide sampling of tissues and different experimental conditions, such as liver, brain, muscle and testis. The ENCODE RNA-seq data covering ovary, mammary gland, stomach, kidney, liver, lung, spleen, colon and heart, are also included in our data. Co-expression patterns in these data can be highly informative for co-functionality. We assigned isoform-level expression values for each of these experiments using the state-of-the-art tools [32], [34], [35] (**Figure 1**). Public RNA-seq datasets came from different experimental protocols and such information is not always recorded in databases in a standardized way. We filtered these datasets based on their quality and coverage (see Methods). This data collection serves as our genomic data input. Essentially, each isoform was described by a vector of values specifying its normalized expression value in various RNA-seq samples. Gene Ontology is arranged in a hierarchy, where complex relationships exist between different terms. To test different variations of MIL and tune the parameters of our algorithms, we focused on a list of biological process terms that have been voted by biologists to be able to describe and cover different biological processes that are experimentally testable [55]. This list included 99 terms, with GO term size 20–300.

**Figure 2:**
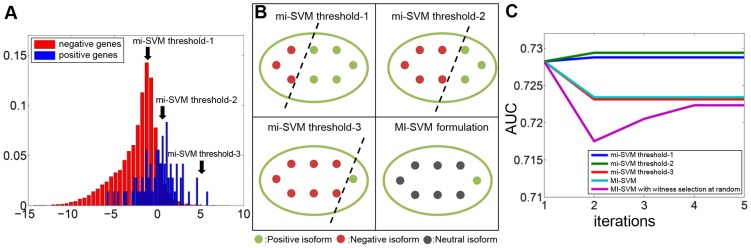
Performance comparison of different formulations of the SVM-MIL algorithm in predicting isoform functions. A. The histogram shows the score distribution of the instances in the positive bags and the negative bags in the training set. Different threshold choices in mi-SVM are based on the distribution of scores of negative genes. The first threshold is equal to the mode of distribution of scores from negative instances in the training set. The second threshold is equal to the 75% percentile of scores of the negative instances in the training set. The third threshold is equal to the maximum score of negative instances in the training set. B. This panel illustrates how different thresholds and formulations can divide the isoforms in a positive bag into positive, negative and neutral classes. Three thresholds in mi-SVM represent different degrees of strictness for assigning labels. The first threshold is the least strict, which assigns most of the isoforms from positive genes as positive, whereas the third threshold is the strictest, which in general leaves only one positive instance in every positive bag. For the MI-SVM formulation, only one isoform per positive gene is assigned as positive, and other isoforms are dropped (*i.e.* neutral class). C. Performance comparison of three different threshold choices for the mi-SVM formulation, the MI-SVM formulation and the MI-SVM formulation with random witness selection. This plot shows that the mi-SVM formulation with threshold-2 performs best in terms of AUC.

Two important parameters (other than the standard SVM parameters) in this algorithm are the proportion of positive isoforms to be labeled as positive in each iteration, and whether we should label the rest of isoforms in the positive bag negative or discard them. Therefore, we tested two basic formulations of the algorithm. The first approach tries to impute all non-witness instances in positive bags as negative instances and then considers the problem as a supervised learning problem. The second approach tries to identify a single witness from each positive bag which is responsible for the positive label. Then, a classifier is built based on these witnesses only, while other instances are dropped. SVM formulations of these two approaches are, respectively, mi-SVM and MI-SVM [51]. For the first approach, we envisioned that different ratio of instances can be retained as ‘witnesses’ and tested three different cutoffs (**Figure 2A–B**).

Ideally, testing of isoform function prediction should use isoform-level gold-standard functional annotation. However, such comprehensive functional annotation does not exist in any database (if they did exist, regular supervised classification would be sufficient to predict isoform functions). Therefore, we first evaluated the performance of our algorithms at the gene level. The probability of each gene to be associated to a function is assigned with the maximum value of all its instances, under the assumption that the eventual gene function is carried out by at least one of its isoforms. For all methods and parameters tested, the algorithm converged within several iterations. Additionally, we found that different thresholds or methods resulted in relatively stable performance on the gene level. mi-SVM with 75% of all negative scores as the cutoff for defining ‘witnesses’ resulted in the highest AUC of 0.73 (**Figure 2C**). Therefore, we used this method for inferring isoform functions and all the evaluation and validation below is based on this threshold.

#### Cross-validation of the function prediction algorithm

We adopted several lines of validation to test our algorithm, including computational validation of multi- and single-isoform genes, literature evidence for top predicted candidates and experimental validation of top predictions. For all the following evaluations, we presented 1792 biological process terms with 20 to 300 genes annotated to each. This size range was selected based on previous statistical studies showing that GO terms of this size show robust cross-validation behavior [56].

**Figure 3:**
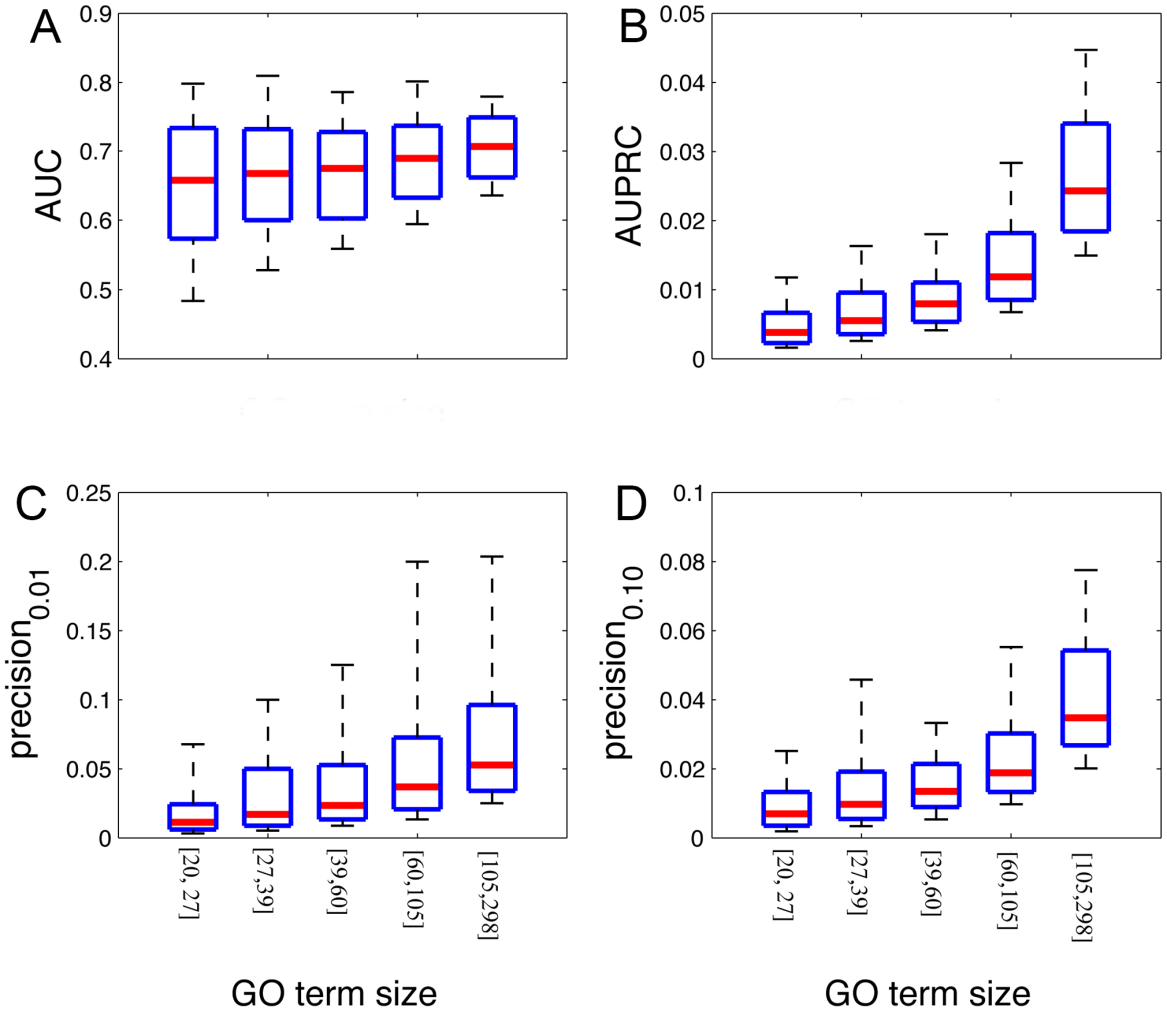
Robust performance of our algorithm to predicting functions using RNA-seq data. We carried out five-fold cross validation to test the performance of our algorithm. For each function, the prediction value for each gene is assigned the maximum prediction value of all of its isoforms, under the assumption that at least one of its isoforms should carry out the function. Because the number of known genes of each GO term systematically affects the prediction performance, we group these terms into 5 groups according to their GO term sizes. (A)–(D) shows the distribution (10, 25, 50, 75, 90%) of the AUCs, the AUPRCs, the precisions at 1% recall and the precisions at 10% recall, respectively.

We first compared the performance of our algorithm in capturing gene-level functions to a direct SVM model using the same data input at the gene level. For each GO term, we carried out five-fold cross-validation to evaluate our prediction results. We partitioned the training and test groups by genes instead of isoforms to prevent information leak in the evaluation process. We used both the area under the ROC curve (AUC) and the area under the precision-recall curve (AUPRC) to measure predictive performance (complete evaluation results are included in Dataset S2). Because GO terms of different sizes vary in terms of predictability [9], we divided all GO terms into 5 groups so that each group contains roughly the same number of GO terms according to GO term size. This resulted in groups of [20, 27], [27, 39], [39, 60], [60, 105] and [105, 298]. For each GO term group, we calculated AUC, AUPRC, precision at 1% recall and precision at 10% recall (**Figure 3**). The median AUCs of the 5 GO term groups are 0.66, 0.67, 0.68, 0.69, and 0.71, respectively. Strictly, these AUC values cannot be directly compared to those reported results in the literature because of the difference in the data used. However, it is still meaningful to benchmark, at least roughly, our results against the reported results. The work of Peña-Castillo *et al.* [57] is a benchmark of the mouse gene function prediction performance in 2008, using heterogeneous genomic data including physical interaction, protein domain, phenotypes and expression. Peña-Castillo *et al.* reported a median AUC in predicting novel gene annotations across 72 GO biological function terms is 0.69 with a standard deviation 0.071. Thus, our algorithm achieved satisfactory results using transcriptomic data alone; the additional benefit of differentiating isoform functions will be detailed in the following sections.

For some of the biological processes, interpreting data at the isoform level can dramatically improve the prediction performance over gene level results. These biological processes are those likely to be affected or carried out by certain specific isoforms of the genes. For example, for GO terms GO:0019882 (antigen processing and presentation), GO:0019395 (fatty acid oxidation), GO:0031032 (actomyosin structure organization), GO:0046649 (lymphocyte activation), GO:0002252 (immune effector process), GO:0045058 (T cell selection) (**Figure S2**), AUPRC increased from 0.087 to 0.105 (20% improvement, baseline 0.0018), from 0.026 to 0.036 (36% improvement, baseline 0.0021), from 0.047 to 0.060 (28% improvement, baseline 0.0012), from 0.0414 to 0.0593 (43% improvement, baseline 0.0101), from 0.037 to 0.046 (27% improvement, baseline is 0.0061), from 0.017 to 0.039 (137% improvement, baseline is 0.0011) respectively using our iterative algorithm compared to using the gene-level SVM method only. The improvement in performance is likely due to having access to more data and eliminating noisy, non-predictive patterns from positive class which is achieved by the MIL formulation.

### Better performance for multi-isoform genes than single-isoform genes

**Figure 4:**
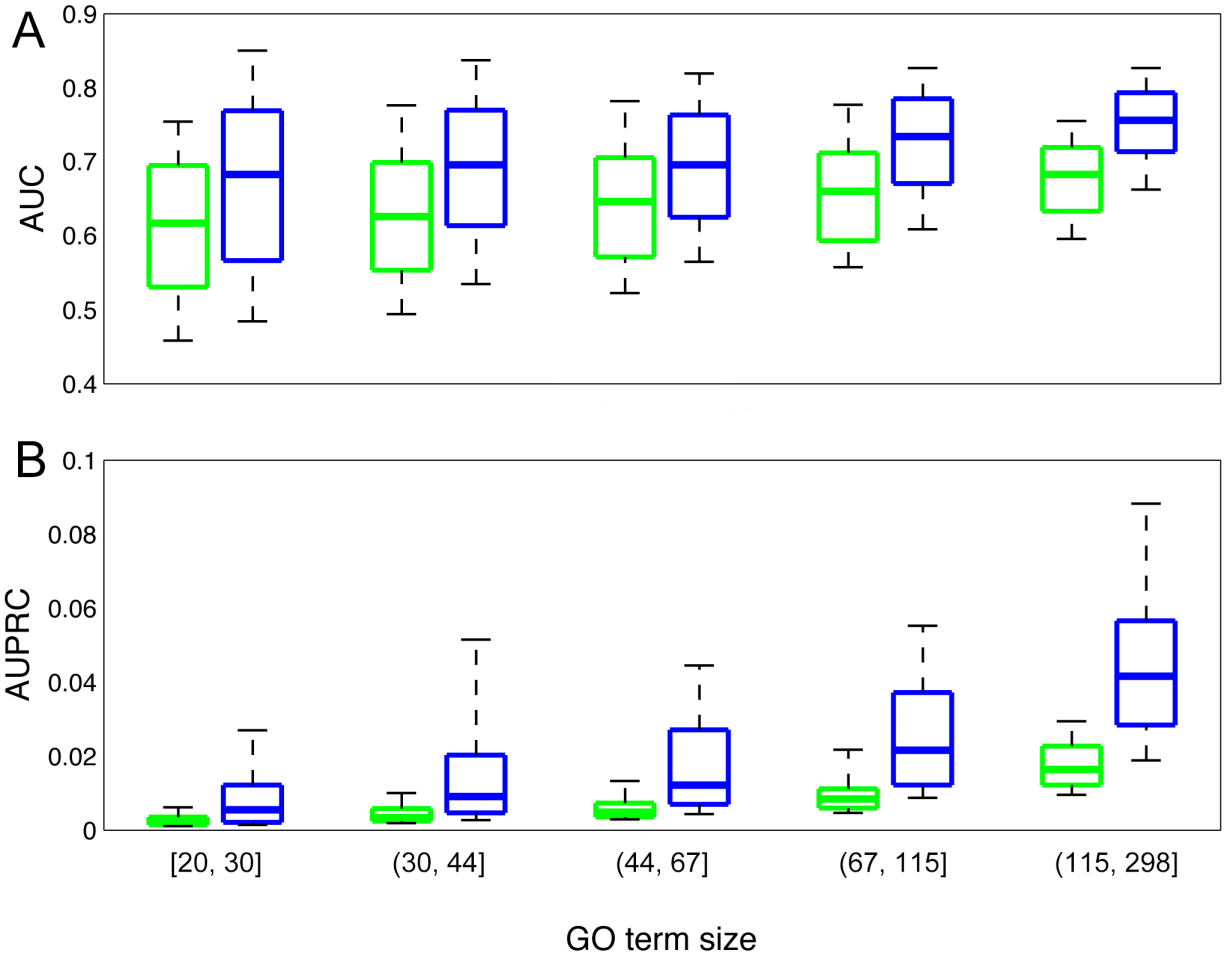
Prediction performance comparison of single-isoform genes (green) and multi-isoform gene (blue) based on AUC (upper panel) and AUPRC (lower panel). We separately evaluated its prediction performance for single-isoform genes and multiple-isoform genes. Two-fold cross-validation was carried out to ensure enough examples in both groups. To ensure comparability, the negatives were randomly selected to ensure that the ratios of positive to negative genes for the multi-isoform group and the single isoform group are the same for each GO term. GO terms were grouped according to the number of genes in the test set. Shown in the box-plot are the AUC (**A**) and AUPRC (**B**) at 10, 25, 50, 75 and 90 percentile, respectively.

The performance obtained in the previous section is a mix between single-isoform genes and multiple-isoform genes. We hypothesize that, if our framework does bring in discriminative power at the isoform level, multi-isoform genes should be predicted with better accuracy than single-isoform genes for the same set of GO terms evaluated. Single-isoform genes include both real ones and those that are missed in the database [58]. In fact, although it has been estimated that 95% of the multi-exon genes in human have multiple isoforms [13], only 13% of the genes are documented with validated multiple isoforms in NCBI. For these genes, the performance is expected to be poorer since the input features for each gene approximate the average of all its isoforms and our algorithm is not applicable to these genes in differentiating isoform functions. Therefore, we separately evaluated the performance of single-isoform genes and multiple-isoform genes. Two-fold cross-validation was carried out to evaluate our models to ensure there are sufficient genes in both the training and test sets for multi- and single-isoform genes. To make the comparison feasible, negatives in each group were randomly chosen to ensure that the ratio of positives to negatives is the same for multi- and single-isoform genes for the same GO term. In doing so, the AUC, AUPRC, precision at 1% recall and precision at 10% recall of each GO term are recalculated for both sets separately. These results are again organized into 5 groups, based on the number of positive genes in the test set for each GO term (**Figure 4**).

**Figure 5:**
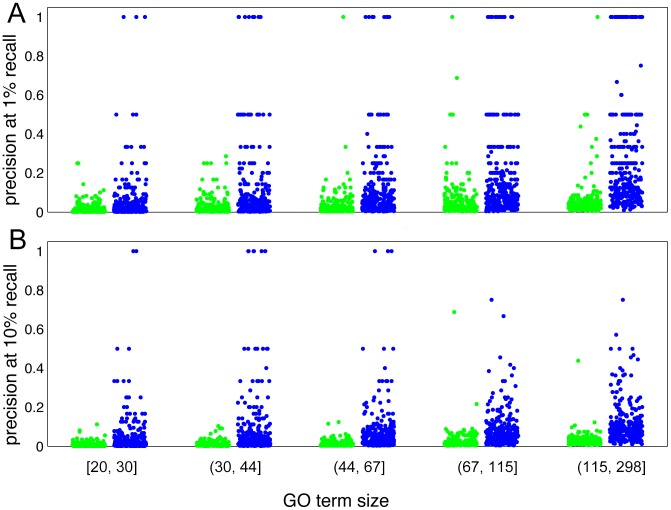
Prediction precision between single-isoform genes (green) with multi-isoform gene (blue). Two-fold cross-validation was carried out to ensure that enough examples are included in both the single-isoform group and the multi-isoform group. The negatives were randomly selected to ensure that the ratios of positive to negative genes for the multi-isoform group and the single isoform group are the same for each GO term, so that the baseline precision for each GO term is equal for the two groups. GO terms were grouped according to the number of genes in the test set. Each dot represents the precision value of an individual GO term. A. Precision at one percent recall. B. Precision at ten percent recall.

We found consistently better performance for multi-isoform genes than single-isoform genes for the same GO term evaluated (**Figure 4**; complete evaluation results are included in Dataset S3). For multi-isoform genes, we acquired median AUCs of 0.68, 0.70, 0.70, 0.73 and 0.76 for the five groups of multi-isoform genes, compared to median AUCs of the same groups of GO terms for single-isoform genes, 0.62, 0.63, 0.65, 0.66 and 0.68, which correspond to 57%, 56%, 34%, 47% and 40% improvements against the baseline (0.5), respectively. Similar better performance is seen for AUPRC values. We observed an increase from 0.002 to 0.006 (162% improvement, baseline is 0.0009), from 0.003 to 0.009 (168% improvement, baseline is 0.0015), from 0.005 to 0.012 (152% improvement, baseline is 0.0021), from 0.008 to 0.022 (156% improvement, baseline is 0.0034), from 0.016 to 0.042 (154% improvement, baseline is 0.0072). In fact, 71% of the 1591 GO terms with more than 20 positive genes and less than 300 positive genes have gained better performance for multi-isoform genes. This implies that our approach is effective in capturing the functionality of multi-isoform genes, which is robust regardless of the metrics used to quantify performance. For example, for GO terms GO:0000279 (M phase), GO:0006952 (defense response), GO:0006936 (muscle contraction), GO:0007507 (heart development), GO:0002252 (immune effector process), GO:0003002 (regionalization), precision at 10% recall increased from 0.121 to 0.571 (baseline 0.0069), from 0.051 to 0.500 (baseline is 0.0103), from 0.052 to 0.364 (baseline is 0.0038), from 0.045 to 0.389 (baseline is 0.0097), from 0.042 to 0.750 (baseline is 0.0048), from 0.074 to 0.320 (baseline is 0.0089), respectively. (**Figure S3**). Such precision improvement is robust to accuracy measurement across the entire precision-recall spectrum and consistent across a wide sampling of GO terms. For the five groups of GO terms, median of precision at 1% recall increased from 0.005 to 0.014 (baseline is 0.0009), from 0.008 to 0.031 (baseline is 0.0015), from 0.011 to 0.045 (baseline is 0.0021), from 0.016 to 0.083 (baseline is 0.0034), from 0.028 to 0.154 (baseline is 0.0072) (**Figure 5**). This result indicates that our algorithm is more effective in predicting functions for genes with multiple isoforms than single-isoform genes, which is likely caused by the power of our algorithm in differentiating isoform functions for the same genes.

### Robustness of predictions with respect to gene expression levels and exclusion of homolog gene pairs

**Figure 6:**
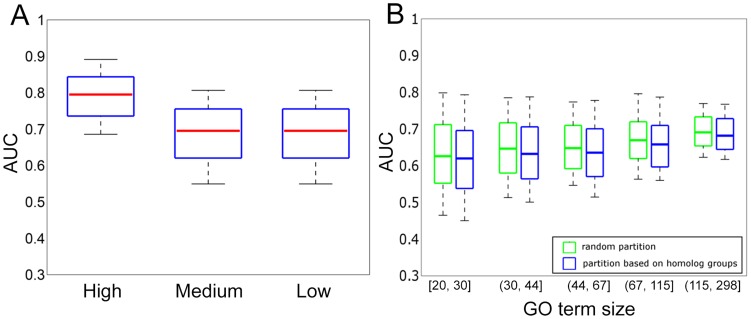
Robust performance of our algorithm in predicting isoform functions. A. Genes are grouped according to their expression levels averaged across all samples in our RNA-seq data collection. The distribution of the performance in AUC across all GO terms is plotted using box-plot. B. The performance in AUC across all GO terms by partitioning the genes according to homologous groups between the training and the test set is compared against the performance of partitioning the genes randomly.

We further considered two factors to validate the robustness of our algorithm. First, because the estimated expression levels are less reliable for genes that have low expression values, we tested whether our algorithm can predict the functions for low-expressing isoforms. We partitioned genes into three groups of equal numbers - the high, the medium and the low groups - based on their expression level averaged across all experimental conditions and evaluated prediction results separately for these groups to see if performance is affected by the overall expression level of genes. We showed that prediction performance is fairly robust to the genes' expression levels (**Figure 6A**). Although the genes in the highest expression group do show better performance (AUC = 0.79), AUCs for the medium (0.69) and low (0.69) groups are comparable to the global accuracy (**Figure 6A**), indicating that our method is applicable to genes that have relatively low expression levels.

Secondly, we tested whether our good performance comes from the homolog gene pairs of which one member is grouped into the training set and the other member is grouped into the test set. This can potentially cause overfitting by information leak, which is a common phenomenon in all functional prediction algorithms, especially those based on sequence information. In order to test robustness of our method with respect to having homolog pairs in test and training sets, we partitioned all genes into equal size test and training sets by putting all genes in a homolog group as defined in Ensembl [59] together and re-evaluated the performance. We found that the performance is comparable to the case where paralog genes can occur in training and test sets (**Figure 6B**). This suggests that our method utilizing RNA-seq data integration is also robust to the information leak from homologs.

### Validation of predicted ‘functional’ isoform(s)

To generate the final isoform function prediction, we applied bootstrap bagging to assign scores for each isoform (**Figure 1**, http://guanlab.ccmb.med.umich.edu/isoPred/ for complete prediction results). The median AUC for the bootstrap result across all GO terms is the same as the cross-validation result, indicating the robust performance of bootstrapping. Essentially, genes are sampled with replacement to construct a training set, and the rest of the examples form an “out-of-bag” set. This process is iterated, and the eventual predictions are drawn from the median across all “out-of-bag” sets. Bootstrap bagging is suitable for our isoform function prediction task, where the numbers of positive and negative examples are highly imbalanced [56]. The robustness of bootstrap bagging has been tested for positive example set sizes ranging from less than 20 to more than 200 [56], [60], which is close to the GO term sizes for which we provide predictions in this study.

Because each GO term has a different background probability for a gene to be associated with it, we calculated the fold change against background for each gene to be associated to a GO term. Indeed, it is pervasive that isoforms of the same gene are assigned with different confidence levels for the same function under consideration (see **Table S1** for a selection of examples and http://guanlab.ccmb.med.umich.edu/isoPred/ for complete prediction results). Because biological functions of transcripts are eventually delivered at the protein level, we hypothesized that the functional isoform(s) of a gene must be expressed at the protein level in the normal physiological condition. We therefore used splice variant protein expression data in normal mammary tissue to validate our predictions. Using our previously developed protocol [61], we identified genes of which only one isoform is strongly expressed (but the other isoforms are not detected). In this gene list, we focused on the ones that have a known specific function (*i.e.*, the function has less than 300 genes annotated to them), but their isoforms are predicted with drastically different confidence levels to carry out this function. In total this resulted in 15 isoform groups. We could then check whether the predicted ‘responsible’ isoform(s) correspond to the expressed isoforms in normal breast tissues.

We found a strong match between the expressed splice variant and the isoform(s) predicted to be responsible for the known specific function of the gene (**Table 1** **and Dataset S4**). All the expressed isoforms are predicted with the highest value in at least one of the known functions, indicating the consistency between the predicted functional isoforms and the expressed isoform. For example, we predicted that NM_172745.3 of Tufm is responsible for its function translation (34 fold over background probability), while the other isoform, NM_001163713.1, is much less likely to be functional (3 fold over background probability). Indeed, only NM_172745.3 is expressed in normal breast tissue. Among the six alternatively spliced isoforms of Tardbp, we correctly predicted that NM_145556.4 is the one responsible for its function in RNA splicing and stabilization; this variant is the only one identified in the proteomic sample. In fact, of the 9 functions annotated for Tardbp, NM_145556.4 is predicted with the highest value eight times. The majority of the exceptions occur for tissue or developmental-stage-specific GO terms, such as GO:0007507 heart development, GO:0035051 cardiac cell differentiation and GO: 0006936 muscle contraction. The predicted functional isoform is not the one identified in our proteomic sample, most likely because our sample is tissue-specific and normal and these functions might be carried out by other isoforms in other tissues or conditions. However, overall, the predicted functional isoforms are consistent with the ones we identified in our proteomic data, indicating that our algorithm can correctly identify the functional splice variants in normal conditions.

**Table 1:** Examples for predicted functional isoforms that are validated using proteomic data.

Gene Name	Identified transcript in proteomic data	GO term ID	GO term name	Fold change of prediction score
Tufm	NM_172745.3	GO:0006412	translation	34.10
Tardbp	NM_145556.4	GO:0006396	RNA processing	10.48
Tardbp	NM_145556.4	GO:0008380	RNA splicing	15.08
Tardbp	NM_145556.4	GO:0016071	mRNA metabolic process	6.15
Tardbp	NM_145556.4	GO:0043487	regulation of RNA stability	3.28
Tardbp	NM_145556.4	GO:0043488	regulation of mRNA stability	7.00
Tardbp	NM_145556.4	GO:0043489	RNA stabilization	7.50
Tardbp	NM_145556.4	GO:0048255	mRNA stabilization	6.70
Tardbp	NM_145556.4	GO:0051817	modification of morphology or physiology of other organism involved in symbiotic interaction	3.08
Ola1	NM_025942.2	GO:0006163	purine nucleotide metabolic process	2.23
Ola1	NM_025942.2	GO:0006195	purine nucleotide catabolic process	2.20
Ola1	NM_025942.2	GO:0006200	ATP catabolic process	2.92
Ola1	NM_025942.2	GO:0009141	nucleoside triphosphate metabolic process	2.53
Ola1	NM_025942.2	GO:0009143	nucleoside triphosphate catabolic process	2.32
Ola1	NM_025942.2	GO:0009144	purine nucleoside triphosphate metabolic process	2.60
Ola1	NM_025942.2	GO:0009146	purine nucleoside triphosphate catabolic process	2.38
Ola1	NM_025942.2	GO:0009154	purine ribonucleotide catabolic process	2.59
Ola1	NM_025942.2	GO:0009166	nucleotide catabolic process	2.08
Ola1	NM_025942.2	GO:0009199	ribonucleoside triphosphate metabolic process	2.29
Ola1	NM_025942.2	GO:0009203	ribonucleoside triphosphate catabolic process	2.24
Ola1	NM_025942.2	GO:0009205	purine ribonucleoside triphosphate metabolic process	2.42
Ola1	NM_025942.2	GO:0009207	purine ribonucleoside triphosphate catabolic process	2.66
Ola1	NM_025942.2	GO:0046034	ATP metabolic process	3.55
Ola1	NM_025942.2	GO:0046700	heterocycle catabolic process	2.08
Ola1	NM_025942.2	GO:0072521	purine-containing compound metabolic process	2.35
Ola1	NM_025942.2	GO:0072523	purine-containing compound catabolic process	2.10
Myom1	NM_010867.2	GO:0003012	muscle system process	21.02
Lmna	NM_001002011.2	GO:0007517	muscle organ development	3.04
Lmna	NM_001002011.2	GO:0014706	striated muscle tissue development	4.03
Lmna	NM_001002011.2	GO:0042692	muscle cell differentiation	4.69
Lmna	NM_001002011.2	GO:0051146	striated muscle cell differentiation	7.05
Lmna	NM_001002011.2	GO:0055001	muscle cell development	4.57
Lmna	NM_001002011.2	GO:0060537	muscle tissue development	5.35
Lmna	NM_001002011.2	GO:0061061	muscle structure development	4.28
Gpx3	NM_008161.2	GO:0006518	peptide metabolic process	2.16
Gpx3	NM_008161.2	GO:0006749	glutathione metabolic process	2.25
Gpx3	NM_008161.2	GO:0042743	hydrogen peroxide metabolic process	2.18
Gpx3	NM_008161.2	GO:0044106	cellular amine metabolic process	2.14
Gpx3	NM_008161.2	GO:0072593	reactive oxygen species metabolic process	2.38

### Validation of predicted disparate functions for isoforms of CDKN2a and of ANXA6

Our algorithm is more than just identifying the ‘functional’ isoform of a gene. The power of our algorithm to predict the functional disparity between isoforms is further illustrated by isoforms that carry out different aspects of gene functions. It is relatively common that only one isoform is predicted to be responsible for one particular function of a gene, as described in the previous section. In this section, we focus on analyzing specific examples in which isoforms of a single gene are assigned with unrelated biological functions.

**Figure 7:**
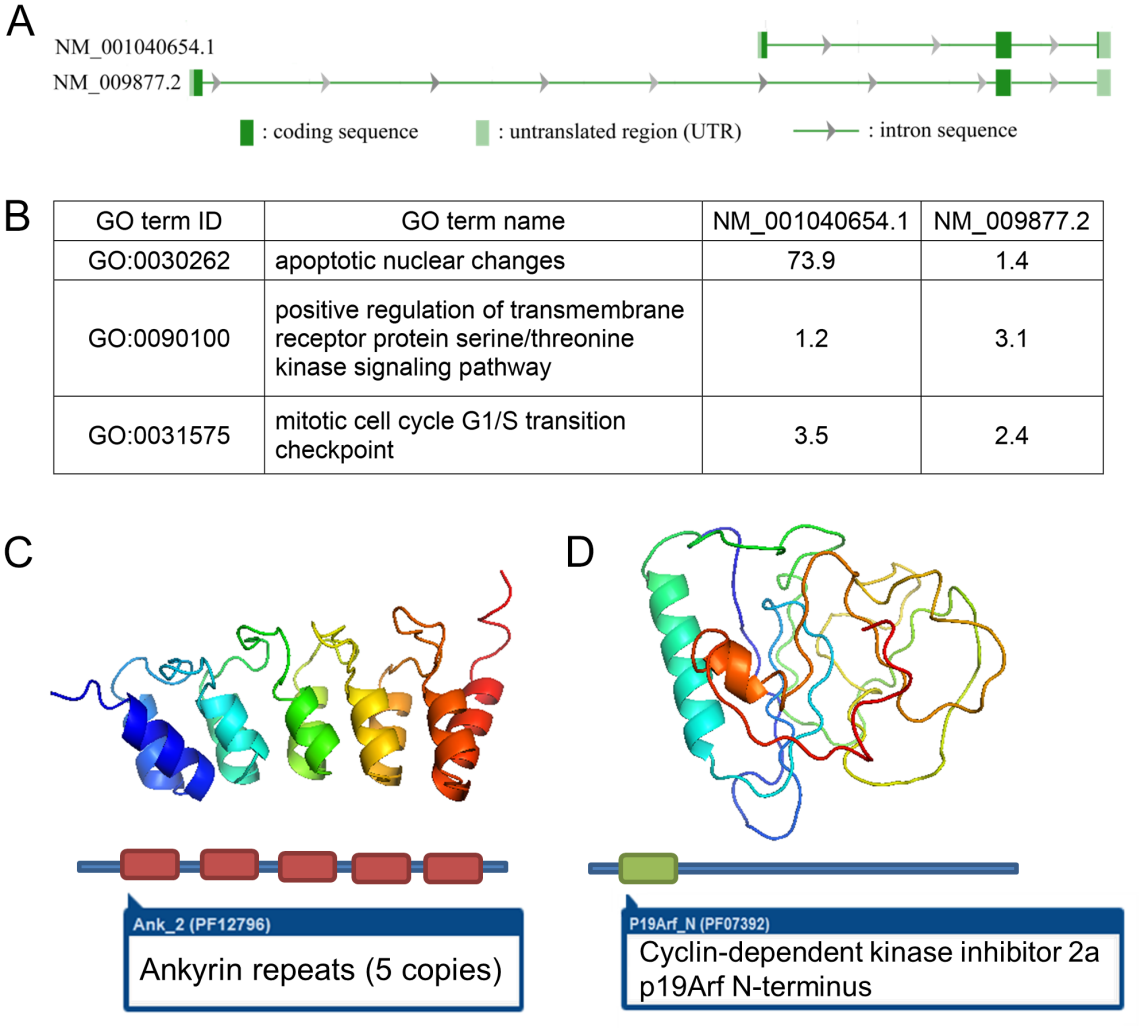
Predicted functions for isoforms of CDKN2a and their predicted protein structures. A . Gene model for NM_001040654.1 and NM_009877.2. **B**. Predicted functions for NM_001040654.1 and NM_009877.2. **C**. The computationally modeled structure of NM_001040654.1 is characterized by five ankryin repeats. **D**. The modeled structure of NM_009877.2 has a CDKN2a N-terminus domain.

CDKN2a is the only known example where alternative splicing results in different reading frames (**Figure 7A**). Two genes (XBP1, GNAS1) produce alternate reading frames, but start with single transcript and therefore do not fall into the realm of alternative splicing. We predicted that the two isoforms of CDKN2a would carry distinct functions for the gene (**Figure 7B**). NM_001040654.1 is predicted to be involved in apoptotic nuclear changes with a probability 74 times the background probability, while the probability for NM_009877.2 approximates background. On the other hand, NM_009877.2 is predicted to be involved in positive regulation of the transmembrane receptor protein serine/threonine kinase signaling pathway (3 times background), while NM_001040654.1 is not. Because crystallized protein structures are available for both proteins in the human but not in the mouse, we used I-TASSER, the state-of-the-art protein structure prediction algorithm [62], to model the 3-D protein structures of the two isoforms (**Figure 7C–D**). Although the translated products of NM_001040654.1 (168 aa) and NM_009877.2 (169 aa) are almost the same lengths, the transcript sequences are in different open reading frames. This resulted in five ankyrin repeats in the NM_001040654.1 (**Figure 7C**), compared to a cyclin-dependent kinase inhibitor N-terminus domain in NM_009877.2 (**Figure 7D**). The drastically different 3-D structures support the potential disparate functions predicted by our algorithm.

The function predictions for NM_009877.2 and NM_001040654.1, which we made by mining only large-scale public RNA-seq datasets, are consistent with the two distinct biological roles of the two isoforms. NM_009877.2 is an inhibitor of CDK4 kinase, a member of the Ser/Thr protein kinase family, directly supporting its role in GO:0071900, regulation of protein serine/threonine kinase activity. NM_001040654.1 encodes an alternate open reading frame (ARF) that generates a protein structurally unrelated to NM_009877.2. This protein enhances p53-dependent transactivation and apoptosis [63], supporting its role in apoptotic nuclear changes. Interestingly, although coding for structurally dissimilar proteins, both isoforms share a common functionality in cell cycle G1 control [64]. This shared functionality is correctly predicted by our algorithm; for regulation of G1/S transition of mitotic cell cycle (GO:2000045): NM_001040654.1 has a probability 3.5 times the background probability, and NM_009877.2 has a probability 2.4 times that of background.

The CDKN2a example involves isoforms of drastically different protein domains. We used the isoforms of ANXA6 (NM_013472.4 and NM_001110211.1) to validate our model in predicting isoforms of very similar structure. The only difference between the two isoforms at the protein sequence level is the presence of six residues in the longer NM_013472.4 (‘VAAEIL’, 525–530) which are missing in NM_001110211.1. The three-dimensional structures of the translated sequences of these isoforms were published by some authors of this paper [65]. Although the global topology of the I-TASSER models for the two isoforms of ANXA6 is almost identical (with RMSD = 0.38 Å and TM-score = 0.99), there is an obvious structural variation identified by TM-align [66]. The positions of Thr-535 and Ser-537 in NM_013472.4 compared to NM_001110211.1 make NM_013472.4 more likely to undergo phosphorylation [65]. The fold changes on GO-terms related to phosphorylation by our function prediction algorithm supported the conclusion from the structural comparisons. The fold change for peptidyl-serine phosphorylation for NM_013472.4 was 3.5 compared to 1.9 for NM_001110211.1. Re-searching the mass-spectrometric data with phosphorylation on Serine or Threonine (Phospho (S) and Phospho (T)) as potential residue modification, yielded a peptide ‘DQAQEDAQVAAEILEIADTPSGDKTSLETR’ (found only in NM_013472.4) with 3281.506 daltons as the mh (calculated peptide mass plus a proton) indicating potential phosphorylation [67]. In contrast, the peptide that matched the spliced region in NM_001110211.1 (residues ‘VAAEIL’ are missing in this peptide) did not show any phosphorylation. These observations further supported our predictions. In addition, the overall function enrichment showed the smaller isoform, NM_001110211.1 as involved in biological processes related to cell adhesion and cell migration, whereas the longer form is predicted to be involved in localization. It is important to emphasize the fact that our computational predictions based on RNA-seq data alone were able to pick up the differences between these two isoforms with 99% sequence identity and predict distinct functions for the isoforms. These findings suggest that our approach can solve the pressing need of isoform function differentiation, which would be invaluable for a better understanding of the diversity of functions created by alternative splicing of a limited set of genes.

## Discussion

Gene functions are delivered through alternatively spliced transcript isoforms that encode proteins of different functions. It is highly beneficial that the investigation of functions is carried out at the isoform level. From this point of view, the standard gene function prediction paradigm has a major drawback in that it considers a gene as one single entity without differentiating its isoforms. The availability of transcript-level expression data from RNA-seq provides a rich resource for addressing this drawback. However, algorithmically, any supervised learning algorithm developed for gene function prediction cannot be directly applied to isoform function prediction because of the lack of isoform-level, ‘ground-truth’ functional annotations.

To address this challenge, we developed an iterative algorithm that predicts functions at the individual isoform level by conceptualizing a gene as a ‘bag’ of isoforms of potentially different functions. Our key idea is to iteratively extract the common pattern of a subset of isoforms across the positive genes of the function under investigation, aiming at maximizing the coherence within this subset of isoforms and the discriminative power against the other ‘negative’ genes (genes not related to the specific function under consideration).

Through experimental validation, we demonstrated that our approach in combination with publicly available RNA-seq data is capable of differentiating isoform functions, promising better and deeper understanding of gene functions. RNA-seq data are the richest resource for genome-wide, isoform-level data so far. But the basic concept is extendable to other large-scale datasets providing isoform-level information, such as protein domain data and post-translational regulation datasets. These datasets are not included in this study due to their strong overlap with the ‘Gold-Standard’ gene ontology annotations, which might lead to a circularity problem in evaluating our algorithms. Furthermore, our study only focuses on the base learner SVM. However, our approach is highly extendable to other modeling methods, such as logistical regression and random forests.

Our study is limited to the incomplete isoform catalog maintained by NCBI, but it can be readily updated whenever the genome annotation of isoforms is updated. Additionally, alternatively spliced isoforms often show tissue-specific expression and functions [23], [27], [68]–[71]. Our generic algorithm does not yet take the tissue-specific functionality into consideration. We expect that more accurate and biologically meaningful isoform function prediction could be achieved if tissue specificity were taken into account. As a result, our validation carried out in breast tissue is only used to validate the ‘generic’ functions of the isoforms. Recent studies found that the same principal isoform is often present in different tissues [72]–[75]. We expect that tissue-specific functions can be validated in corresponding tissues when these tissue-specific predictions can be made. Our study is further limited by the current technology to assign isoform-level expression values, as well as the differential capability between platforms for capturing isoform-specific expression. We used Cufflinks in the Tuxedo suite [32], one of the state-of-the-art algorithms, to estimate the isoform-level read counts and achieved good performance. However, if more advanced algorithms are developed, our algorithm could directly utilize the estimates from those algorithms and generate isoform function predictions.

Our approach represents a novel and generic strategy to look at gene functions at a higher resolution. Cross-validation, literature, and experimental analysis of proteomic data provided evidence that our algorithm is powerful in differentiating isoform functions. Broadly speaking, the genomic data integration field typically relies on the supervised learning concept, which cannot generate predictions for spliced isoforms, *e.g.*, predicting gene-disease association and gene regulatory networks. We envision that similar concepts will be developed for generating isoform-level models for these prediction tasks.

## Methods

### Pre-processing public RNA-seq datasets

We downloaded 811 RNA-seq experiments for the mouse from the NCBI sequence read archive (SRA) database as of May 1, 2012 [30]. These datasets represent different conditions and tissues. Heterogeneity of the datasets allowed us to look at the isoform expression variations across different conditions and tissues. Because datasets are heterogeneous in terms of library preparation procedures and sequencing platform (Dataset S1), we adopted the following processing and filtering pipeline to ensure that all datasets included in the final predictions have sufficient coverage. NCBI build 37.2 reference genome was downloaded from the TopHat homepage, and BOWTIE2 [76] index files were created by bowtie2-build software. For each RNA-seq dataset, short reads were aligned against the NCBI *Mus musculus* reference genome (Build 37.2) using TopHat v2.0.051 [32], [34]. The reference GTF annotation file from NCBI (build 37.2, downloaded from TopHat homepage) was given to TopHat and the no-novel-juncs option was used. With this option, TopHat creates a database of splice junctions indicated in the supplied GTF file and maps the previously unmapped reads against the database of these junctions to create an estimate of isoform expression levels. Then we used Cufflinks v2.0.0 [32] to measure the relative abundances of the transcripts using normalized RNA-seq fragment counts [32], [35]. The unit of expression levels is Fragments Per Kilobase of exon per Million fragments mapped (FPKM). To ensure data quality and overall coverage, we removed those experiments with less than 10 million reads or with less than 50% reads being successfully mapped to the genome. The above procedure resulted in 365 experiments used in our study. Genes detected in less than half of the experiments were removed. After filtering these poorly-covered genes, there are 19209 genes left with a total of 24274 isoforms. Distribution of the number of isoforms per gene is included in Figure S4. FPKM values were log_2_-transformed; missing values were approximated with a value of “-15”.

### Assembling gene-level gold standard functional annotations

We constructed gold standard gene functions using the Gene Ontology (GO) database [48], [77]. For each biological process term, we treated the genes which are annotated to that GO term and any of its descendent terms as positives and others as negatives. We maintained all GO evidence codes. The sources of these annotations include (1) hand annotation from primary literature, (2) electronic annotation based on gene name and symbols, (3) annotation from SwissProt keywords and (4) Enzyme Commision (EC) numbers [78]. These annotation sources are reasonably accurate for our analysis.

### Mathematical definition and solution of the isoform function prediction problem

As stated in the Results section, the isoform function prediction problem is a Multiple Instance Learning problem. Since the introduction of MIL by [79] for the drug activity prediction, several methods to solve this problem have been proposed in the literature. In [80], the authors introduced the concept of Diversity Density, whose aim is to find a point in feature space that has a high Diverse Density. This means high density of instances from positive bags and low density of instances from negative bags. Additionally, Ray and Page [81] proposed the method multiple-instance regression. Their algorithm assumed that each bag has a witness instance and treated it as a missing value; then the EM (Expectation-Maximization) method was used to learn the witness instances and do the regression simultaneously. Ramon et al. [82] utilized the Neural Network technique on Multiple Instance Learning and proposed the Multiple Instance Neural Network. Finally, Andrews et al. [51] applied the Support Vector Machines to Multiple Instance Learning.

In this paper, we chose to use a method that utilizes the Support Vector Machine. Two different approaches have been proposed to solve the MIL problem using SVM. The first tries to impute all non-witness instances in positive bags as negative examples and then considers the problem as a supervised learning problem. The second tries to identify a single witness from each positive bag which is responsible for positive label. Then, a classifier is built based on these witnesses only, while other instances are dropped out of the classification process. SVM formulations of these two approaches are labeled mi-SVM and MI-SVM by Andrews et al. [51]; we implemented and tested several alternatives of these algorithms.

Without loss of generality, we assume that the *i*th gene with *m* isoforms is denoted as 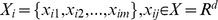. The corresponding class label *y_i_* is set to 1 if the gene is annotated to the function under consideration and *0* if not. For each gene, we hypothesize that if a gene is annotated to a function, at least one of its isoforms should be annotated to the function; if a gene is a negative example used in training, none of its isoforms can be annotated to the function, *i.e.*,
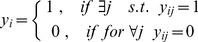
(1)


Then, the mi-SVM formulation of MIL can be written as follows
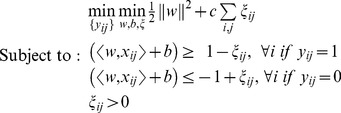
(2)


In the standard classification setting, the labels *y_ij_* of the isoform *x_ij_* would be given; however, in equation (2) labels of isoforms that belong to a positive gene are treated as unobserved hidden integer variables. Therefore, the soft-margin criterion is maximized jointly over hyperplanes and over all possible label assignments. The algorithm is looking for a separating hyperplane such that all isoforms of negative genes are in the negative half-space, whereas there is at least one isoform from every positive gene in the positive half-space. Meantime, the margin is maximized with respect to the selected labels.

Alternatively, in the MI-SVM formulation, the definition of margins is extended to bag-level. Margin of a bag with respect to a separating hyperplane can be defined as the maximum margin of its instances. In the case of positive bags, bag margin is defined by the most positive instance, whereas, for negative bags, the bag margin is defined by the least negative instance. Using this definition of bag margin, the MI-SVM formulation can be written as follows:
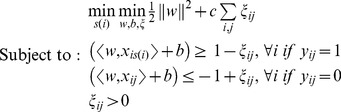
(3)where *y_i_* takes the form 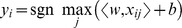 and *s(i)* is the selector variable that denotes the isoform selected as witness from each positive gene. Note that, for the mi-SVM formulation, every instance in a positive bag has an effect on the margin maximization equation, whereas, in the MI-SVM formulation, only one instance per positive bag is taken into account, because this instance alone will determine the margin of the bag.

Finding the optimum solution to (2) and (3) is a combinatorial optimization problem, which cannot be found efficiently with the state-of-the-art tools. Therefore, we approximated the solution by using the following optimization heuristics which is proposed by Andrews *et al.* [51]. Both formulations of MIL explained above can be considered as mixed-integer problems. In the mi-SVM formulation, instance margin is maximized over hidden labels of instances in positive bags, whereas, in the MI-SVM formulation, bag margin is maximized over selector variable, which selects a single witness from every positive bag. Optimization heuristic uses the fact that, given these integer variables, the problem reduces to a quadratic programming problem which can be solved exactly. The optimization heuristic includes two steps: (i) for a set of given integer variables (i.e. hidden labels in mi-SVM and selector variable in MI-SVM), solve the soft-margin maximization problem and find the optimal separating hyper-plane, (ii) for a given separating hyper-plane, update all integer variables so that they maximize the objective locally. These two steps are run iteratively until integer variables are not updated anymore in step (ii). The following workflow explains this optimization heuristic further in detail for both formulations.

1. Initialization: Initially, we assign all instances (isoforms) in positive bags (genes) as positives, *i.e.*,
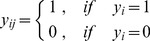
(4)where *y_i_* = 1 if the gene is annotated to the function under consideration, and *y_i_* = 0 if otherwise.
2. Loop:
  (2.1) Model building: construct a maximum margin classification model using positive and negative instances. Using this model, we calculate a prediction score for all instances (including those instances in positive bags which are not “witnesses”) in the training set.
  (2.2) Integer variable updating:
  In the mi-SVM formulation, instances in each positive bag are assigned to a label based on prediction score calculated in the previous step. One can choose different thresholds for scores to partition instances into positive and negative classes. Here, we investigated following three thresholds and chose the second threshold, since it gave the best performance (**Figure 2**)
    (i) The first threshold is equal to the mode of the distribution of scores from negative instances in training set.
    (ii) The second threshold is equal to the 75% percentile of scores of all negative instances in training set.
    (iii) The third threshold is equal to the maximum score of negative instances in the training set.
  These three thresholds represent different degrees of strictness for assigning labels. The first threshold is the least strict; it assigns most of the instances from positive bags as positive, whereas the third threshold is the most strict, generally leaving only one positive instance in every positive bag.
  In the MI-SVM formulation, we chose only the instance with the maximum score in each positive bag as the “witness”; the remaining instances from this bag are not assigned to any class. (*i.e.* dropped out of the margin calculation).
  Note that in mi-SVM every instance in positive bags is assigned to either positive or negative, but in MI-SVM only one instance per positive bag is assigned positive and other instances from the same bag are discarded.
  (2.3) Stop criterion checking: When the assignment of integer variables does not change anymore (*i.e.* label assignments of instances in positive bags for mi-SVM, witness selector variable for MI-SVM), or the assignment of integer variables reverts to one of the assignments in previous iterations, go to Step (3); otherwise go back to step (2.1).
3. Ending iteration: use the model built in the last iteration to predict all instances. At the gene level, the score of each gene is assigned as the maximum score of all its isoforms.

### Estimation of probability score for isoforms using bootstrap bagging

For every function, we need to assign each isoform a score no matter whether the gene that the isoform belongs to has an annotation or not. We therefore used bootstrap bagging to estimate the probability that an isoform is associated with a specific biological process. Essentially all genes are sampled with replacement (0.632 bootstrap) to construct a training set. The scores of the held-out set are recorded and the process is iterated 30 times. For each isoform, the final score is assigned with the median across all iterations.

### Proteomic data processing

Functions of splice variants must eventually be delivered at that protein level. To test whether the predicted differential functions are correct, we compiled the data from LC-MS/MS of normal mammary tissue [83]. The original study reported that normal tissues were harvested from 5 normal mice, processed into tissue lysates and pooled. The pooled sample was digested by trypsin for mass spectrometric analysis. The mzXML files were searched against our modified ECgene database for alternative splice variant analysis using X!Tandem [84].

### Web implementation

All prediction results are stored in MySQL databases and delivered through a searchable website: http://guanlab.ccmb.med.umich.edu/isoPred.

## Supporting Information

Dataset S1 **Input RNA-seq dataset description.**
(XLSX)

Dataset S2 **Five-fold cross-validation results.**
(TXT)

Dataset S3 **Separated evaluation for genes annotated with multiple isoforms and a single isoform.**
(TXT)

Dataset S4 **Comparison between expressed splice variant in the normal breast cell sample and predicted ‘functional’ isoforms.**
(XLSX)

Figure S1 **Differences between the traditional gene function prediction problem and the isoform function prediction problem.** We use maximum margin as a base learner to illustrate the differences between a traditional classification problem for gene function prediction and our scheme for predicting isoform functions. **A**. Traditionally, a gene is treated as one single entity. The positive examples, defined as genes annotated to a specific function, are separated from the negative examples (other genes) by an SVM classifier. **B**. A single gene may contain several isoforms of which only some carry out the function under investigation. Genes here are considered as ‘bags’, each of which may contain one to several isoforms, defined as ‘instances’. A positive gene must have at least one of its isoforms carrying out the function under consideration. None of the isoforms of a negative gene can carry out the function under study. The hyperplane trained to separate the positive isoforms and negative isoforms must satisfy the above criteria.
(TIF)

Figure S2 **Comparison of gene-level prediction performance resulting from gene expression data (dashed green) and isoform expression data (solid blue).** For each GO term-specific gold standard, we developed models using gene-expression data with standard SVM and isoform-expression data with our prediction framework, respectively, and compared their precision recall curves. Shown here are six representative examples, where significant improvements were achieved when using isoform-expression data and our iterative learning strategy.
(TIF)

Figure S3 **Precision recall curve comparison between single-isoform genes (dashed green) and multiple-isoform genes (solid blue) for some GO terms**.
(TIF)

Figure S4 **Histogram of number of isoforms per gene according to NCBI annotation file (build 37.2).** This figure shows only multi-isoform genes, single-isoform genes are excluded.
(TIF)

Table S1 **Example isoform groups that are predicted with differential functions.**
(DOCX)
