# Supplementary figures and images for: Systematically Differentiating Functions for Alternatively Spliced Isoforms through Integrating RNA-seq Data
Source: PLoS Comput Biol. 2013 Nov 7;9(11):e1003314. doi: 10.1371/journal.pcbi.1003314 (PMC3820534; doi:10.1371/journal.pcbi.1003314)

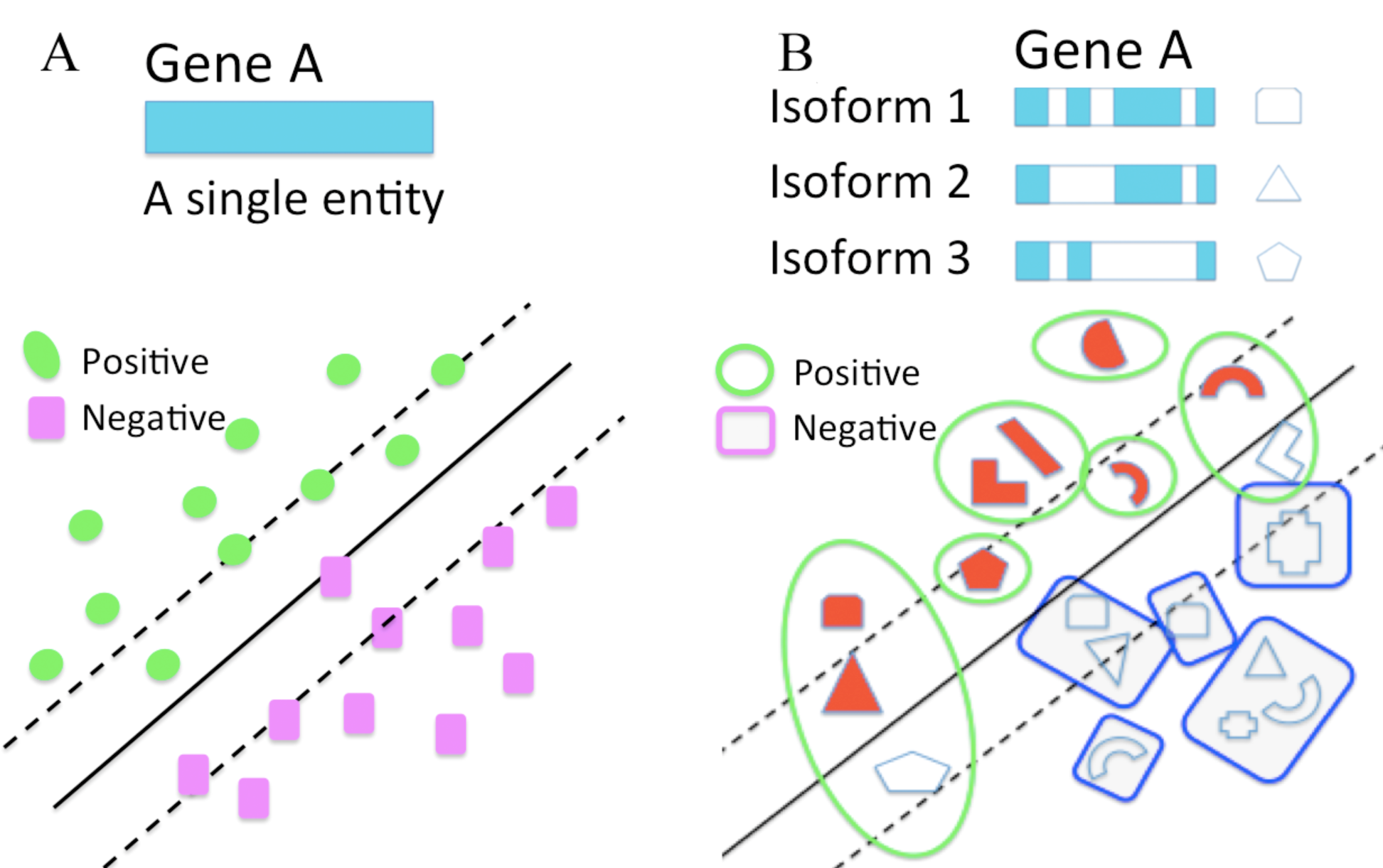

Supplement: Figure S1 — Differences between the traditional gene function prediction problem and the isoform function prediction problem. We use maximum margin as a base learner to illustrate the differences between a traditional classification problem for gene function prediction and our scheme for predicting isoform functions. A. Traditionally, a gene is treated as one single entity. The positive examples, defined as genes annotated to a specific function, are separated from the negative examples (other genes) by an SVM classifier. B. A single gene may contain several isoforms of which only some carry out the function under investigation. Genes here are considered as ‘bags’, each of which may contain one to several isoforms, defined as ‘instances’. A positive gene must have at least one of its isoforms carrying out the function under consideration. None of the isoforms of a negative gene can carry out the function under study. The hyperplane trained to separate the positive isoforms and negative isoforms must satisfy the above criteria. (TIF) [file pcbi.1003314.s005.tif]

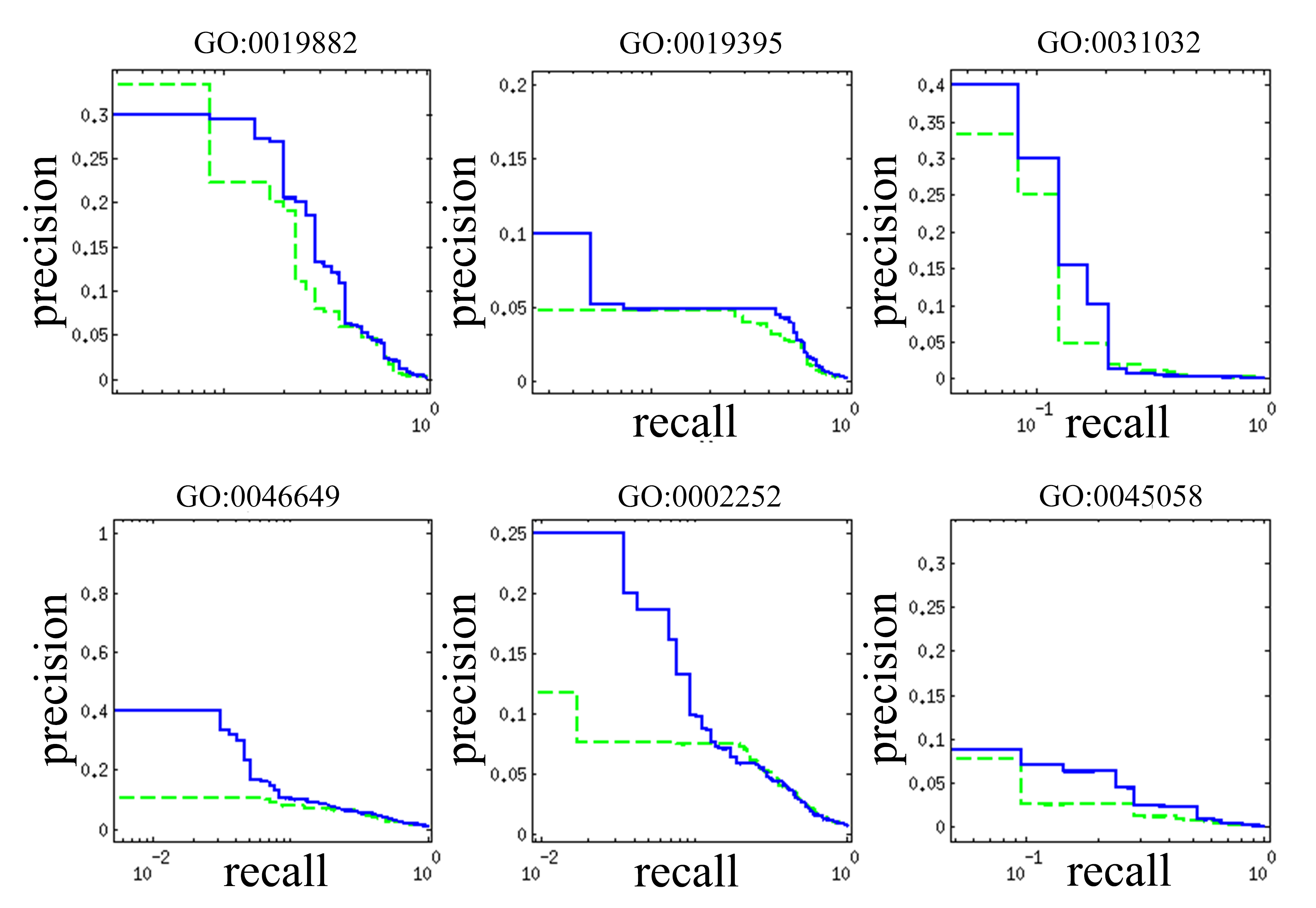

Supplement: Figure S2 — Comparison of gene-level prediction performance resulting from gene expression data (dashed green) and isoform expression data (solid blue). For each GO term-specific gold standard, we developed models using gene-expression data with standard SVM and isoform-expression data with our prediction framework, respectively, and compared their precision recall curves. Shown here are six representative examples, where significant improvements were achieved when using isoform-expression data and our iterative learning strategy. (TIF) [file pcbi.1003314.s006.tif]

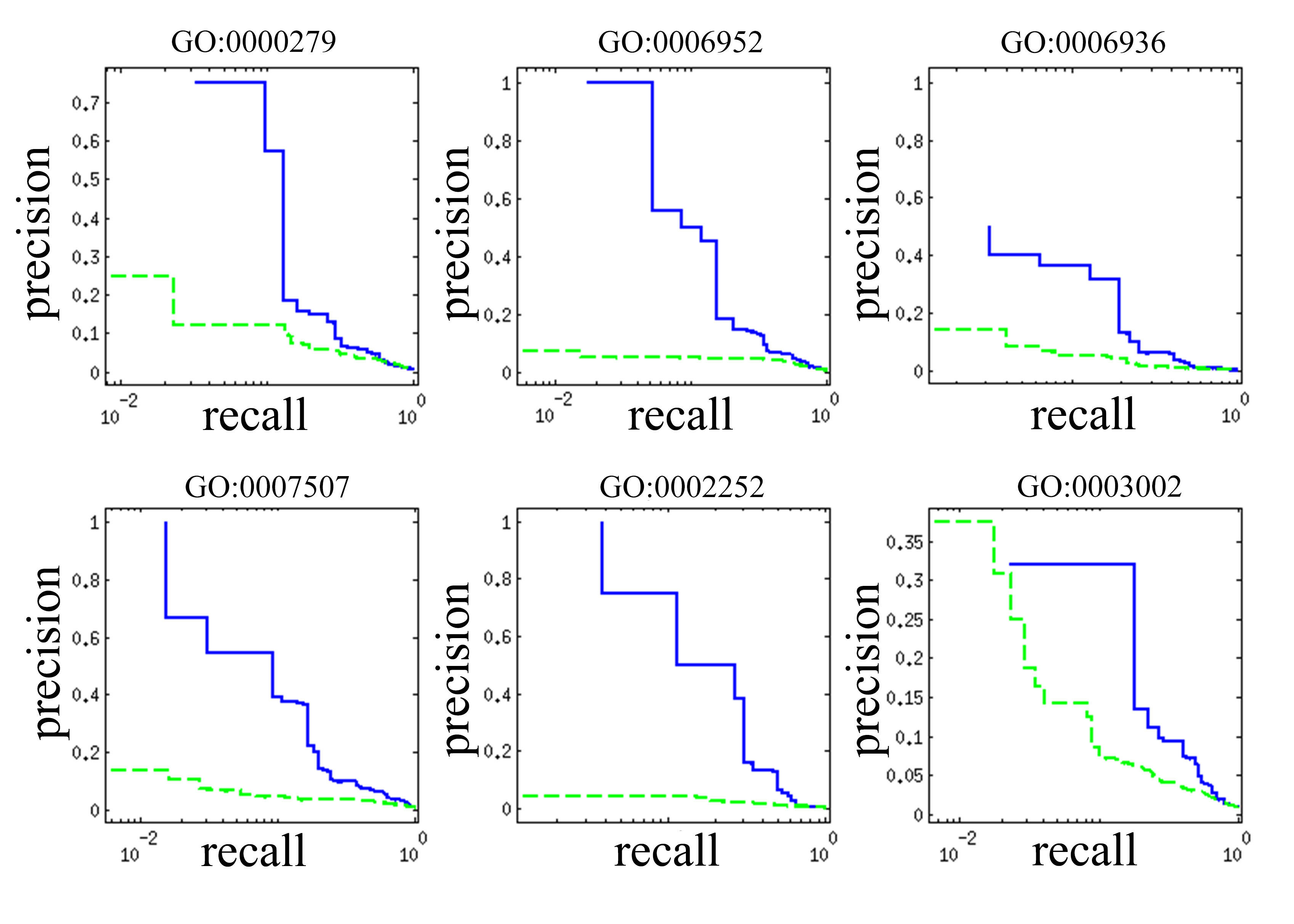

Supplement: Figure S3 — Precision recall curve comparison between single-isoform genes (dashed green) and multiple-isoform genes (solid blue) for some GO terms. (TIF) [file pcbi.1003314.s007.tif]

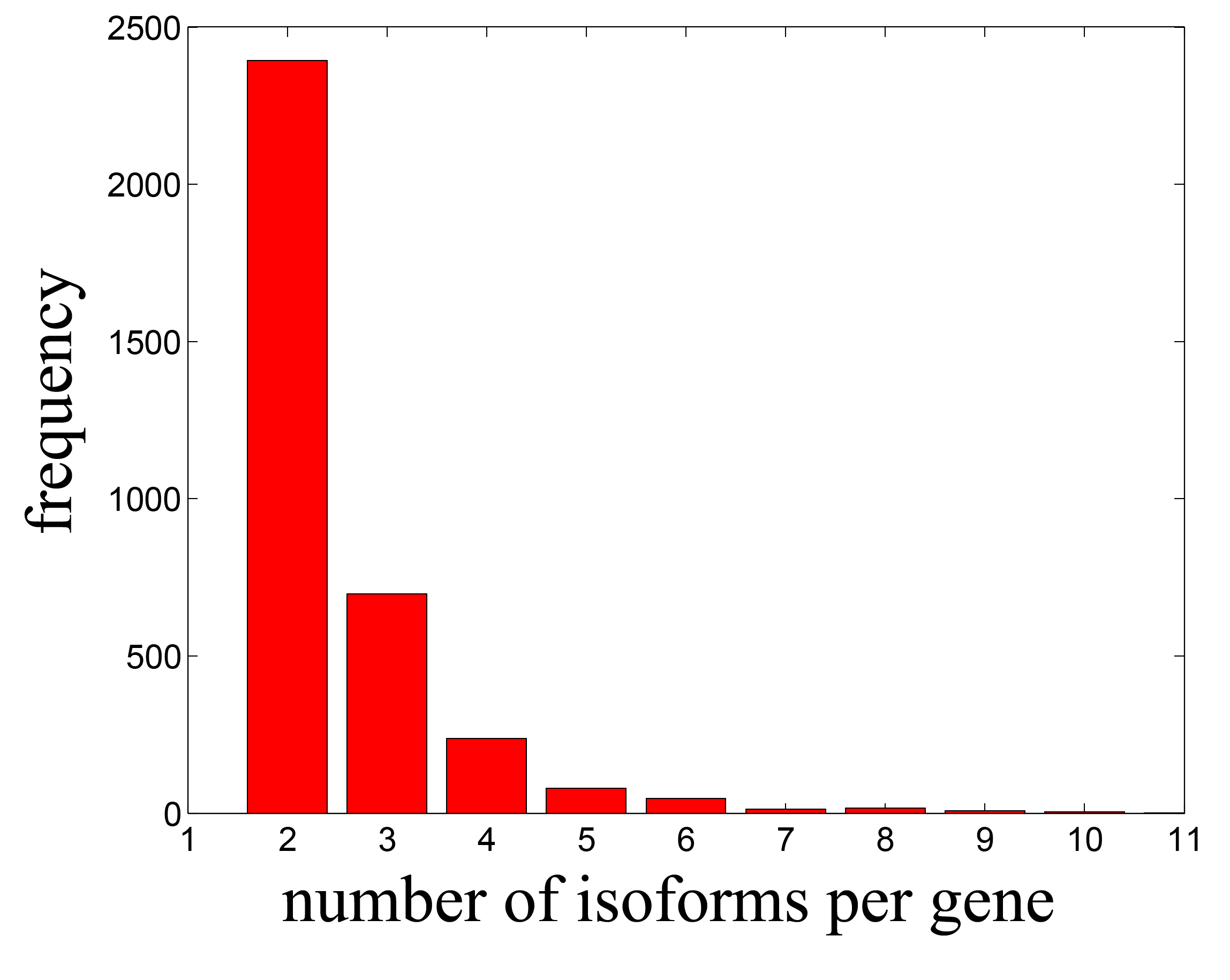

Supplement: Figure S4 — Histogram of number of isoforms per gene according to NCBI annotation file (build 37.2). This figure shows only multi-isoform genes, single-isoform genes are excluded. (TIF) [file pcbi.1003314.s008.tif]
