# Supplementary material for: Systematically Differentiating Functions for Alternatively Spliced Isoforms through Integrating RNA-seq Data
Source: PLoS Comput Biol. 2013 Nov 7;9(11):e1003314. doi: 10.1371/journal.pcbi.1003314 (PMC3820534; doi:10.1371/journal.pcbi.1003314)
Supplement: Table S1 — Example isoform groups that are predicted with differential functions. (DOCX) [file pcbi.1003314.s009.docx]

**Table S1. Example isoform groups that are predicted with differential functionality.**

| Gene name | GO term ID | GO term name | Isoform name | Fold change |
| --- | --- | --- | --- | --- |
| Tpm1 | GO:0031032 | actomyosin structure organization | NM_001164248.1 | 800.37 |
|  |  |  | NM_001164249.1 | 33.95 |
|  |  |  | NM_001164250.1 | 1.92 |
|  |  |  | NM_001164251.1 | 128.06 |
|  |  |  | NM_001164252.1 | 1.92 |
|  |  |  | NM_001164253.1 | 1.69 |
|  |  |  | NM_001164254.1 | 31.87 |
|  |  |  | NM_001164255.1 | 10.00 |
|  |  |  | NM_001164256.1 | 1.69 |
|  |  |  | NM_024427.4 | 2.03 |
| Pde4dip | GO:0030239 | Myofibril assembly | NM_001039376.2 | 1.99 |
|  |  |  | NM_001110163.1 | 98.88 |
|  |  |  | NM_177145.3 | 1.92 |
|  |  |  | NM_178080.4 | 2.19 |
| Rtn4 | GO:0006749 | glutathione metabolic process | NM_024226.4 | 61.92 |
|  |  |  | NM_194051.3 | 1.57 |
|  |  |  | NM_194052.3 | 1.69 |
|  |  |  | NM_194053.3 | 1.49 |
|  |  |  | NM_194054.3 | 1.07 |
| Rtn4 | GO:0022029 | telencephalon cell migration | NM_024226.4 | 1.52 |
|  |  |  | NM_194051.3 | 1.60 |
|  |  |  | NM_194052.3 | 1.72 |
|  |  |  | NM_194053.3 | 1.31 |
|  |  |  | NM_194054.3 | 23.83 |
